# Mathematical and computational modeling in biology at multiple scales

**DOI:** 10.1186/1742-4682-11-52

**Published:** 2014-12-27

**Authors:** Jack A Tuszynski, Philip Winter, Diana White, Chih-Yuan Tseng, Kamlesh K Sahu, Francesco Gentile, Ivana Spasevska, Sara Ibrahim Omar, Niloofar Nayebi, Cassandra DM Churchill, Mariusz Klobukowski, Rabab M Abou El-Magd

**Affiliations:** Department of Physics and Department of Oncology, University of Alberta, Edmonton, Canada; Department of Oncology, University of Alberta, Edmonton, Canada; Department of Physics, University of Alberta, Edmonton, Canada; Department of Mechanical and Aerospace Engineering, Politecnico di Torino, Torino, Italy; Department of Biology, Ecole Normale Supérieure de Lyon, Lyon, France; Department of Chemistry, University of Alberta, Edmonton, Canada; Department of Biological Sciences, University of Alberta, Edmonton, Canada

**Keywords:** Mathematical biology, Epidemiological models, Cellular physiological models, Cancer models, Maximum entropy, Molecular dynamics, Force fields, Solvation free energies, Quantum mechanics, Computational enzymology

## Abstract

A variety of topics are reviewed in the area of mathematical and computational modeling in biology, covering the range of scales from populations of organisms to electrons in atoms. The use of maximum entropy as an inference tool in the fields of biology and drug discovery is discussed. Mathematical and computational methods and models in the areas of epidemiology, cell physiology and cancer are surveyed. The technique of molecular dynamics is covered, with special attention to force fields for protein simulations and methods for the calculation of solvation free energies. The utility of quantum mechanical methods in biophysical and biochemical modeling is explored. The field of computational enzymology is examined.

## Introduction

Mathematical, computational and physical methods have been applied in biology and medicine to study phenomena at a wide range of size scales, from the global human population all the way down to the level of individual atoms within a biomolecule. Concomitant with this range of sizes between global to atomistic, the relevant modeling methods span time scales varying between years and picoseconds, depending on the area of interest (from evolutionary to atomistic effects) and relevance. This review will cover some of the most common and useful mathematical and computational methods. Firstly, we outline the maximum entropy principle as an inference tool for the study of phenomena at different scales, from gene evolution and gene networks to protein-drug molecular interactions, followed with a survey of the methods used for large scale systems—populations, organisms, and cells—and then zooming down to the methods used to study individual biomolecules—proteins and drugs. To study the large systems, the most common and reliable mathematical technique is to develop systems of differential equations. At the molecular scale, molecular dynamics is often used to model biomolecules as a system of moving Newtonian particles with interactions defined by a force field, with various methods employed to handle the challenge of solvent effects. In some cases, pure quantum mechanics methods can and should be used, which describe molecules using either wave functions or electron densities, although computational costs in time and resources may be prohibitive, so hybrid classical-quantum methods are often more appropriate. Quantum methods can be particularly valuable in the study of enzymes and enzymatic reactions.

## Maximum entropy in biology and drug discovery

Two reasoning methods, deduction and inductive inference, have been utilized in the development of theories to interpret phenomena we observe in nature, and to make predictions about complex systems. Deduction allows us to draw conclusions when sufficient information is available, and is contrasted with inductive inference (also known as inductive logic or probable inference). Inductive inference provides a least biased way to reason when the available information is insufficient for deduction. It is called “inference” when we make estimates of quantities for which we do not have enough information to use deductive reasoning, and “induction” when we are generalizing from special cases [1].

When we deal with complex systems, for example either many-body interactions at the microscopic level, complicated regulatory protein-protein networks at the mesoscopic level, or population genetics at the macroscopic level, we never have enough knowledge to completely understand the system. Therefore, we normally rely on inductive inference based on the available information to infer the most preferred solution to problems related to these systems. Particularly, we are interested in a mathematical tool for inductive inference based on the Bayesian interpretation of probability, the rules of probability theory, and the concept of entropy. Bayesian interpretation treats probability as a degree of our knowledge about a system of interest, rather than the frequency of appearance of an event. Cox demonstrated that this type of probability can be manipulated by the rules of standard probability theory [2]. This forms the building blocks of inductive inference, termed Bayesian inference. Moreover, Caticha and Giffin have shown that Bayesian inference is a special case of entropy-based inference [3]. Therefore, our discussion in this section will be founded on entropy-based inference.

First, we briefly address the basics of entropy-based inference, which includes using entropy as an information measure and a tool for inductive inference, then we provide examples in the fields of biology and drug discovery to demonstrate that these fields benefit from the application of inductive inference. Regarding using entropy as an information measure, we consider two examples. The first example provides a clue to investigate genomic evolution through appropriate genomic sequence analysis [4]. The second one discusses robustness of biological networks from information point of view [5]. Regarding using entropy as a tool for inductive inference, we consider another two examples. The first one demonstrates the benefit of introducing this inference in virtual screening for drug discovery [6]. The second one then shows an application of this scheme in fragment-based drug design [7]. These examples also illustrate in a straightforward way how to extract information and unveil the global characteristics of the biological systems. They show that there exists a universal reasoning platform to solve any problem of interest that is independent of the specifics of a given type of problem. The key in this platform lies in the answer to the question, “What are the constraints in the system?” Once the constraints are determined, the maximum entropy principle provides a robust, universal and least biased prescription for information processing. Furthermore, it helps us to analyze problems and gain insights into the functioning of the underlying complex systems.

### Entropy as an information measure

Shannon’s pioneering work on the quantification of information loss during communication established a new viewpoint on entropy, which was until then only known as a measure of randomness in thermodynamics [8]. Since then, using entropy as an information measure has attracted much attention not only in signal processing, but also in the field of biology.

#### Information in genomic evolution

With the advance of genomic sequencing technology there is more and more genomic sequence data available for species across the three domains: Bacteria, Archaea, and Eukaryota. The question is, how do we compare complete genomes and extract useful information from the sequencing data?

To address the question of genome comparison, Chang et al. [4] proposed an entropy-based scheme for complete genome comparison. The foundation of their approach is to define the probability distribution that represents our current state of knowledge regarding the occurrence of different combinations of the four bases in DNA sequences. Chang et al. [4] specified that *k*-mer nucleotides in the sequence encode genetic information, where *k* is an arbitrary number. The occurrence of *k*-mers in a DNA sequence characterizes that sequence. Based on this definition, information in sequences can be quantified with Shannon information. Furthermore, Chang et al. [4] introduced the concept of reduced Shannon information, which is defined as the ratio of the Shannon information of the genome to the Shannon information of random sequences, so as to quantify to what extent the information contained in the genome is different from the information in a random DNA sequence. Note that this concept is similar to the concept of relative entropy, which is discussed in the next section. Based on reduced Shannon information (or relative entropy), a universal feature across three taxonomic domains was observed; namely, the effective root-sequence length of a genome, which is defined as the ratio of genome length and reduced Shannon information, linearly depended on *k*, and was a genome-independent constant. Furthermore, this study revealed a possible genome growth mechanism: at an early stage of evolution, a genome is likely to utilize random segmental duplication, which would maximize reduced Shannon information. These insights not only provide a clue to the origin of evolution but also may shed light on further questions, such as which genes are responsible for drug resistance.

#### Robustness of biological networks

The development of high throughput screening techniques, such as microarray technology, has generated numerous protein-protein interaction data to map out biological networks, and has revealed regulatory mechanisms of the biological entities involved in the networks. Many studies have suggested that the robustness of biological networks may be the key for identifying systems that can tolerate external perturbations and uncertainty triggered by external forces [9, 10]. It has further been shown that the robustness of biological networks shares a global feature; these networks are scale-free networks, which means that one can observe a power-law degree distribution in these networks.

Therefore, there have been many endeavors to provide more insights into the origin of these power-law distributions. Bak et al. [8] proposed the mechanism of self-organized criticality, which leads to a scale-free structure in complicated systems. An entropy-based interpretation described by Dover [5] suggested a promising and intuitive way to understand the emergence of power-law distributions in complicated networks. According to Dover’s studies on a toy model, the emergence of the power-law distributions is merely a consequence of the maximum entropy principle when the internal order of sub-networks of a complicated large network remained fixed. Note that the internal order was defined as the mean of the Boltzmann entropy over all sub-networks. In the framework of entropy-based inference, the power-law distributions of biological networks simply represent the most preferred choice that maintains the fixed internal order of the sub-networks.

### Entropic scheme for inductive inference

In addition to the use of entropy as an information measure, the concept of entropy also plays a role in inductive inference. The inductive inference addressed here refers to two processes. The first process is the determination of the most likely state of knowledge about a system of interest based on the information in hand. The second process is the determination of the most likely updated state of knowledge when we acquire new information regarding the system. The foundation of inductive inference is the maximum entropy principle and relative entropy. The least biased inference one can make based on the information in hand is the one that maximizes the relative entropy of all possible new and old beliefs. For more details, the reader is referred to Caticha [1].

#### Entropy in molecular docking

Our first example of applying entropy for inductive inference is *in silico* drug discovery. Virtual screening has attracted much attention in the pharmaceutical industry [12, 13]. It provides a more economical way to screen diverse chemicals as drug candidates compared with a wet-lab approach. Basically, it consists of the creation of a chemical library, followed by searching optimal ligand-receptor binding modes through docking algorithms, and finally the evaluation of binding affinities. There are three criteria that are required to successfully identify drug candidates. First, the chemical library needs to be large and contain diverse chemical structures. Second, conformational search algorithms need to be able to search possible binding modes within a reasonable time. Third, an appropriate scoring function needs to be utilized to correctly evaluate the binding affinity of the chemical structures. In the framework of information theory, the first and third criteria are the fundamental information required in virtual screening process. The second criterion then can be treated as an information processing guideline. The efficiency and accuracy of this step will depend on the methods of information processing.

Genetic algorithms, which borrow from the concept of genomic evolution processes to search conformations of complex targets and chemical structures, are commonly used in docking protocols, such as AutoDock [14]. Chang et al. have offered a better alternative, MEDock [6]. Although MEDock did not completely exploit entropic-based inductive inference for searching, it does utilize the maximum entropy principle as a guideline to make decisions during this process. The fundamental question asked in MEDock is “What is the probability of finding the deepest energy valley in a ligand-target interaction energy landscape?” Maximum entropy provides a direction to update the initial guess of binding modes (described by an almost uniform distribution) to the optimal mode (a localized distribution around the global energy minimum).

#### Entropy in aptamer design

The second example of entropy for inductive inference is aptamer design. Aptamers are short nucleic acid sequences that are traditionally identified through an experimental technique, the Systematic Evolution of Ligands by Exponential Enrichment (SELEX) [15, 16]. Aptamers can bind to specific molecular targets including small molecules, proteins, nucleic acids, and phospholipids, and can also be targeted to complex structures such as cells, tissues, bacteria, and other organisms. Because of their strong and specific binding through molecular recognition, aptamers are promising tools in molecular biology and have both therapeutic and diagnostic clinical applications [15–18]. Unfortunately, some limitations of SELEX have slowed the progress of discovering specific aptamers for various applications [18]. With the help of entropy-based inductive inference, a fragment-based approach has been developed to design aptamers given the structure of the target of interest [18].

The concept of the fragment-based approach to aptamer design is to ask the question “Given the structural information about the target, what is the preferred probability distribution of having an aptamer that is most likely to interact with the target?” The solution was found using entropy-based inductive inference [7]. This approach initially determines the preferred probability distribution of first single nucleotide that likely interacts with the target. Subsequently, the approach iteratively updates the probability distribution as more nucleotides are added to the growing aptamer. The maximum entropy principle allows us to determine to what extent this update is sufficient, and what is the sequence of nucleotides that is most likely to bind to the target. This method has been applied to design aptamers to bind specifically to targets such as thrombin, phosphatidylserine [19] and galectin-3 (under experimental confirmation).

The maximum entropy principle and inductive inference just provide one reasoning platform to make the most preferable inference based on all kinds of information for understanding biological systems at different scales. In the next section, a variety of mathematical and computational models addressing other aspects that have been developed for biological and medical problems are surveyed.

## Mathematical and computational models for biological systems

In recent years, mathematical biology has emerged as a prominent area of interdisciplinary scientific research. It is not a new area of research, but with recent advances in medical and computational methods, it has grown extensively, being applied to solve many health related problems across a spectrum of life sciences. Areas of mathematical biology where modeling has made contributions to biology and medicine include epidemiology, cell physiology, cancer modeling, genetics, cellular biology, and biochemistry. Because there is such a broad range of topics and methods that can be discussed, we limit ourselves to a discussion of how differential equations have been used to solve important biological problems in epidemiology, cell physiology, and cancer modeling, and briefly discuss some of the clinical advances that have arisen from such efforts. For a more extensive review on mathematical modeling for each of these branches of science, we refer the reader to recent books on these topics [20–23]. For the reader who is interested in learning more about mathematical biology from a beginner’s perspective, books by Edelstein-Keshet [24], Murray [25, 26], and Britton [27] are also recommended.

Here, we highlight only a few of the models that have been developed to study epidemiological, physiological, and cancer problems. The reader is encouraged to look more extensively into the literature regarding other models that have been developed and successfully applied to improve present medical treatments.

### Mathematical models in epidemiology

Epidemiology describes the study of patterns, cause and effect, and treatment of disease within a given population [28]. Here, we provide a brief introduction to epidemiological models used for studying the spread of various types of disease, many of which are outlined in [25, 27]. One of the first models to describe the dynamics of a disease caused by a viral infection is the so-called SIR model, an ordinary differential equation (ODE) model developed by Kermack and Mckendrick in 1927 [29].
123

This model, given by equations (1), (2), and (3) (for all differential equation models we omit initial and boundary conditions for ease in reading), describes the rate of change of the number of susceptible (*S*), infected (*I*), and recovered (*R*) individuals in a population over time, where *β* describes the rate of transmission of disease, and *μ* describes the rate of removal of infected individuals (those that have recovered). An important feature of this model is that it incorporates recovered patients, meaning that an individual can acquire immunity, as is often the case for viral-type infections like influenza and measles. This model, although quite basic, provides important information to health care professionals interested in understanding how severe an outbreak is. For example, from these equations, the basic reproduction number given by
4

describes the average number of secondary infections produced by one infected individual introduced into a completely susceptible environment. High values of *R*_0_, corresponding to high numbers of initially susceptible individuals *S*(0), and/or high disease transmission rates *β*, result in the high probability of an outbreak. In particular, if *R*_0_ is less than 1, the infection will not persist (and will eventually die out), whereas if *R*_0_ is greater than 1, the infection will grow (and there will be an epidemic).

One key assumption of this model is that the total population *N* (*N* = *S* + *I* + *R*) is constant and that there is no death or birth. Many models have since been developed to include such population demographics [30–32], the first being completed by Soper [31] in an attempt to understand the dynamics of measles.

A number of extensions have been made to describe a wider class of infections. For example, the SIRS and SIS models allow for the movement of individuals back into a susceptible class S, meaning there may be no immunity to re-infection [30]. Such models are useful in studying bacterial-type infections like tuberculosis and gonorrhea. Other models, referred to as SEIR and SEIRS models, where “E” stands for a latent class of individuals (exposed but not showing symptoms), can be used to describe a disease where a delayed time of infection may exist [33]. For example, this is often the case with individuals suffering from malaria.

A disadvantage of ODE-based modeling is that it assumes the well mixing of large populations of individuals. Also, such models are deterministic, meaning that the outcome is determined solely on the initial conditions and the parameters that govern the dynamics. For some populations, where contacts and transmission rates between individuals may vary, agent based [34, 35] stochastic [36] or network type [36] models may be more useful. Also, age-structured models [37] may be more appropriate for diseases that depend on age, such as AIDS.

Another disadvantage of ODE-based modeling is that it does not describe the movement of individuals through space. This information is extremely important because a disease may not just spread within a single population, but may spread from one location to another. Examples of models that incorporate spatial dynamics include partial differential equations (PDEs). These models have been used to study the outbreak of rabies in continental Europe in the early 1940s [38], as well as to study the more recent outbreak of the West Nile Virus in 1999 in New York State [39]. Other models used to study the spatial spread of disease include patch models [40]. In the patch model of Lloyd and May [40] the authors consider an SEIR modeling approach. Here, the total population is broken up into subpopulations (patches), where *S*_*i*_, *E*_*i*_, *I*_*i*_, and *R*_*i*_ denote the number of susceptible, exposed (latent), infected, and recovered individuals, in each patch *i*, respectively. The dynamics of each patch are governed by their own similar set of differential equations,
5678

In each patch, all model parameters are the same, except the infection rate *σ*_*i*_, which depends on each connection between patches. Here, *σ*_*i*_ is called the force of infection, and is given by the mass action expression
9

where *n* is the total patch number and *β*_*ij*_ is the rate of infection between patches *i* and *j*.

Mathematical models have influenced protocol in disease control and management. Now, such modeling is part of epidemiology policy decision making in many countries [41]. Some important modeling contributions include the design and analysis of epidemiology surveys, determining data that should be collected, identifying trends and forecasting outbreaks, as well as estimating the uncertainty in these outbreaks.

### Physiological models at the cellular level: enzyme kinetics, ion channels, and cell excitability

The field of physiology is arguably the number one biological field where mathematics has had the greatest impact. Two broad areas of physiology where mathematics has made a profound impact are cell physiology and systems physiology. Here, we focus on cell physiology, and restrict ourselves to the topics of enzyme kinetics, ion channels, and cell excitability. For an excellent review on systems physiology, the reader is referred to Keener and Sneyd [22].

The rate of change of a simple chemical reaction can be described by the law of mass action, a law that describes the behavior of solutions in dynamic equilibrium [42]. That is, for a simple reaction given by
10

where *k*_+_ is the reaction rate constant, the rate of the production of product molecules is given by
11

where [*X*] is the concentration of each species *X* = *A*, *B*, *C*. Equation (11) is commonly referred to as the law of mass action. The above formulation can only be used for the simplest of reactions involving a single step and only two reactants, although extensions are fairly straight-forward and have been developed over the past century to describe more complicated reactions [43]. One example is the model of Michaelis and Menten [44], used to describe reactions catalyzed by enzymes. Given the reaction scheme
12

where *S* is the substrate, *E* the enzyme, *P* the product concentration, and *k*_1_, *k*_-1_, and *k*_2_ are the reaction rate constants, Michaelis and Menten describe this reaction by the following four ODEs,
13141516

Assuming that the substrate is at equilibrium (*ds*/*dt* = 0), one can simplify this system and find explicit solutions. Also, without solving these equations, one can gain useful information about the process. For example, the velocity of the reaction (the rate at which the products are formed) is
17

where *V*_max_ = *k*_2_e_o_ and *K*_*s*_ = *k*_-1_/*k*_1_ (*K*_*s*_ is called the equilibrium constant). Equation (17) is often referred to as the Michaelis–Menten equation. Also, the steady-state approximation simplifies the above system of equations so that we can find explicit solutions [45]. This approximation requires that the rates of formation and breakdown of the complex *c* are essentially always equal (*dc*/*dt* = 0). Further extensions of this model have been developed to describe other types of enzyme activity, such as cooperativity [46] and enzyme inhibition [47], and have had similar success.

Computational systems biology has been creating a series of tools that are useful for application to enzyme kinetics. This is particularly true in the area of parameter estimation where several algorithms have been shown to be useful to enzyme kinetics. The availability of increasingly sophisticated and standardized modeling and simulation software will undoubtedly benefit enzyme kinetics [48].

Biochemical networks are sets of reactions that are linked by common substrates and products. The dynamics of biochemical networks are frequently described as sets of coupled ODEs, similar to those given by equations (13) through (16), that represent the rate of change of concentrations of the chemical species involved in the network [48]. The right-hand side in these ODEs is typically the algebraic sum of the rate laws of the reactions that produce or consume the chemical species (positive when it is produced, negative when consumed). There is formally no difference between a biochemical network and an enzyme reaction mechanism, as both conform to this description. For systems biology studies, it is sufficient to represent each enzyme-catalyzed reaction as a single step and associate it with an appropriate integrated rate law [49]. The systems biologist should be cautioned, though, that mechanistic details may indeed affect the dynamics, as is the case with competitive versus uncompetitive inhibitor drugs [50–52].

The Systems Biology Markup Language (SBML) [53] is a standard format to encode the information required to express a biochemical network model including its kinetics. SBML is based on the Extended Markup Language (XML), which is itself a standard widely adopted on the Internet. After a series of progressive developments, there are now several compatible software packages available to model biochemical networks. Some are generic and provide many algorithms, while others are more specialized. This includes not only simulators [54], but also packages for graphical depiction and analysis of networks [43, 55, 56], and databases of reactions and kinetic parameters [57], to name but a few examples. In some cases these packages can even work in an integrated way, such as in the Systems Biology Workbench (SBW) suite [58].

Another area of cell physiology where mathematical modeling has been used to describe complex molecular-scale dynamics is the study of ion channels. Molecules (both large and small) move back and forth across a cell membrane, to ensure that conditions for homeostasis are met [42]. Some molecules are small enough (and soluble to lipids) to diffuse across the membrane, while others require energy, working against electrochemical gradients between the outside and the inside of the cell [42]. For example, differences in ionic potential across a cell membrane can drive ionic current. The Nernst equation,
18

describes the potential difference *V* across a cell membrane, where *c*_*e*_ and *c*_*i*_ are the external and internal ionic concentration (of a particular ion) respectively, *R* is the universal gas constant, *T* is the absolute temperature, *F* is Faraday's constant, and *z* is the ion’s charge. To determine the ionic current across a membrane, one can write the total potential drop across the cell membrane, *V*_*T*_, as the addition of the Nernst potential *V* to the potential drop due to an electrical current *rI*_*c*_ (where *r* is the resistance), so that *V*_*T*_ = *V* + *rI*_*c*_. Solving for the ionic current we arrive at
19

where *g* = 1/*r* is the membrane conductance. Ions only travel through small pores, referred to as ion channels, and the control of such ionic current is vital for proper cellular function.

A second model for determining the ionic flow across a cell membrane is given by the Goldman–Hodgkin–Katz equation,
20

where *P* is the permeability of the membrane to the ion [59]. Such an equation is derived under the assumption of a constant electric field. The use of either the linear equation (19) or nonlinear equation (20) in defining ionic current is often debated and depends on the underlying properties of the particular cell studied.

Using the fact that we can model the cell membrane as a capacitor (since it separates charge), and knowing that there is no net build-up of charge on either side of the membrane, the sum of the capacitive and ionic currents across a membrane should add up to zero,
21

Here, *C*_*m*_ is the capacitance, *I*_ion_ is the ionic current, and *V* = *V*_*i*_ - *V*_*e*_*.* In 1952, through a series of pioneering papers, Hodgkin and Huxley presented experimental data and a comprehensive theoretical model that fit experimental findings, to describe the action potential across the giant axon of a squid [60–64]. The model given by equation (22) (and based on equation (21)), was awarded the Nobel Prize in Physiology and Medicine in 1963, and is possibly one of the greatest mathematical results in physiology to date:
22

In equation (22), *g*_Na_, *g*_K_, and *g*_L_ describe the sodium, potassium, and leakage conductance (other ionic contributions, including the chloride current, that are small), respectively, *V*_Na_, *V*_K_, and *V*_L_ are their respective resting potentials, and *I*_app_ is a small applied current. Hodgkin and Huxley were able to measure the individual ionic currents, and to use this information to determine the functional forms for each of the conductances.

Much of the work completed on ion channels and cell excitability has been used to study diseases that are associated with malfunction of ion channels. As a result, such channels have become new targets for drug discovery [47]. One example of a disease caused by the disruption of the action potential of cardiac myocytes is cardiac arrhythmia. Certain drugs used in the treatment of arrhythmias, such as lidocaine and flecainide, are sodium channel blockers, and so interfere with open sodium channels. Although these drugs have been used in treatment for cardiac arrhythmias, their exact mode of action is not well understood. Current computational models are being developed to understand the function of these, as well as other anti-arrhythmia drugs [65]. Another example of a disease caused by the disruption of ion channels is cystic fibrosis (CF), which has been found to be associated with malfunctions in chloride channel operation [66]. Although there is still no cure for CF, new directions for treatment protocols are being developed [67].

### Models of cancer growth and spread: avascular tumor growth, tumor-induced angiogenesis, and tumor invasion

The primary mathematical modeling techniques used in the area of cancer modeling, include the study of avascular tumor growth, angiogenesis, and vascular tumor growth (tumor invasion), and so in what follows we limit ourselves to these topics.

Growth and development of a solid tumor occurs in two stages: avascular growth and vascular growth (leading to tumor invasion), and has been studied extensively in a biological framework [68]. The modeling of avascular tumor growth is one of the earliest approaches taken by mathematicians to study cancers [69]. The simplest type of model that can be used to describe how cancer cells of a solid tumor change over time is the exponential growth law, given by equation (23).
23

Such an equation is limited in its application, since it suggests that tumors grow (with growth rate *r*) to a tumor of unbounded size. Other models, such as the logistic growth equation (24)
24

have been used to describe tumor size saturation (a type of volume constraint). In particular, equation (24) describes the rate of change of cancer cells *N* in a tumor, where *r* is the growth rate and *k* is the carrying capacity (the maximum number of cancer cells within the tumor).

A limiting case of the logistic equation is the Gompertz model, given by equation (25).
25

This model is one of the most commonly used tumor growth models to date, and was first used by Casey to fit real tumor growth in 1934 [70]. Such ODE-based models are useful because they can be easily analyzed. Also, these models have the advantage that they can be expanded to incorporate other cell types (such as proliferating, quiescent, and dead cells) by the inclusion of more ODEs, one for each cell type. Since these models are similar to the compartmental SIR model described in the section concerning epidemiology, they have similar limitations, one being a lack of spatial information (such as the location of each cell type within a tumor). Some models for solid tumor growth have included such information by incorporation of reaction-diffusion type equations, like equation (26). This latter model, studied by Murray [26], was developed to describe the spatio-temporal invasion of gliomas. Equation (26) is read as follows: cancer cells *C* grow exponentially with rate *ρ,* and diffuse at a rate that depends on whether cells are moving in white brain matter or gray brain matter (diffusion given by *D*(*x*)).
26

Other models by Cruywagen *et al.*[71] and Tracqui *et al*. [72] have used a more realistic logistic growth term for cancer cell growth. However, the survival times calculated for these models are only slightly different from those calculated using model (26), and so using either logistic growth or exponential growth is appropriate in this modelling framework. One limitation of the Cruywagen *et al.* [71] and Tracqui *et al.* [72] is that they are constructed under the assumption of constant diffusion, thus neglecting to distinguish between grey and white matter in the brain. Simulation of models similar to (26), such as those completed by Swanson *et al.*[73], show that incorporation of a spatially dependent diffusion coefficient *D*(*x*) produces images that are in good agreement with real images of gliomas produced by magnetic resonance imaging (MRI).

Other spatial models, like that of equation (27), describe the movement of certain chemical signals, such as growth factors (GFs), which dictate directions for tumor growth [74, 75].
27

In equation (27), the first term on the right-hand side describes the diffusion of the concentration of GFs denoted by *C*, while the second term describes the degradation of *C* with rate *γ*. The third term describes production of *C* at a rate *σ* by a source *S*(***r***). The source is a function of the tumor radius ***r***. Some models incorporate integro-differential equations that describe the radius of tumors over time [75], providing a type of spatial information for each cell type within the tumor. For example, the nutrient concentration often dictates where each type of cell is most likely located within the tumor. Thus, in a radially-symmetric cell, there are typically zero nutrients located at the center of the tumor, and so cells there are dead, defining a necrotic core, whereas the outermost layer is generally nutrient rich, and so it is composed of proliferating cells. Other models, such as those incorporating systems of PDEs, are used to describe the spatial movement over time of proliferating and quiescent cancer cells in the presence of GFs [76].

Typically, solid tumors initially grow to about 2 mm in size. To grow larger, tumors require nutrients. Such nutrients are additionally acquired through the process of angiogenesis, the formation of blood vessels that connect the tumor to the circulatory system [68]. The switch to this angiogenic stage can occur due to multiple factors, many of which are outlined in Semenza [77]. Tumor-induced angiogenesis, caused by the release of GFs or tumor angiogenic factors (TAFs) from the tumor, promotes the growth of endothelial cells (EC), which make up the linings of capillaries and other vessels. ECs then migrate towards the TAF source by chemotaxis. In other words, blood vessels grow towards the tumor.

The first model (given by equation (28)) to describe chemotaxis in a biological system using PDEs was developed by Keller and Segel [78]. Such an equation was initially developed to describe the chemotactic movement of slime molds towards an attracting source called cyclic adenosine monophosphate (cAMP):
28

In the context of cancer cells, the first term on the right-hand side of equation (28) describes random motion (diffusion) *D* of a cell population *u*, while the second term corresponds to chemotaxis of the cells *u* towards a GF. The chemotactic effect is given by *χ* and *υ* is the concentration of the GF. The dynamics of the growth factor is typically modeled by reaction-diffusion equations similar to that given above in equation (27).

Many other models, typically PDE-type models, have extensions similar to those described above, incorporating not only the key interactions of the endothelial cells with angiogenic factors, but also the macromolecules of the extracellular matrix [79, 80]. For an excellent review on mathematical approaches to studying tumor-induced angiogenesis we refer the reader to Mantzaris, Webb, and Othmer [81].

After a tumor has progressed to the stage of angiogenesis, by successfully recruiting blood vessels, the tumor eventually becomes vascular, thus connecting itself to the circulatory system [68]. Once this happens, tumor cells have the ability to enter the circulatory system, depositing themselves at secondary locations within the body and possibly promoting the growth of a secondary tumor, a process referred to as metastasis [68]. As soon as this occurs the cancer has progressed to the point where it is nearly impossible to cure the patient. One of the key steps in the progression to metastasis is the degradation of the extracellular matrix (ECM) by matrix-degrading proteins deposited by cancer cells, and the movement of cancer cells through the ECM. Newer mathematical approaches, using integro-PDEs, have been able to capture the qualitative movement of such cells through the ECM [82–84]. One such model was proposed by Hillen [82].
29

Here*, p*(***x***, *t*, *v*) describes the density of tumor cells at location ***x***, time *t* > 0, and velocity *v* in *V* = [*v*_min_, *v*_max_]. The advection term on the left-hand side of equation (29) describes directed movement of cells along collagen fibers in the ECM with speed *v*. The right-hand side describes changes in the cell’s velocity due to changes in the matrix orientation *q*(***x***, *t*, *v*)*,* where *w* is an appropriate weighting function*.* This matrix can change over time due to cuts made by matrix degrading proteins. For ease in reading we do not give the evolution equation for the matrix distribution *q*.

Many different cancer treatment modalities are available, including the administration of chemotherapeutic drugs and their combinations [85] and radiation treatment [86]. Also, as is often the case with avascular tumors, the solid tumor may be surgically removed, if discovered early enough. With the advancement in imaging techniques, much work has been done to extend earlier modelling of glioma to better model tumor growth as it applies to treatment (removal and radiation). In particular, better models for predicting survival rates have been developed [87], as well as models that predict efficacy of radiotherapy for individual patients [88]. Typically, MRI is used to detect tumors, and provides the imaging information needed for validating mathematical models. Other imaging techniques, such as Diffusion Tensor Imaging (DTI) (a technique which measures the anisotropic diffusion of water molecules in a tissue), have been used to better establish diffusion parameters required for models to predict the appropriate boundary of a tumor. Such information can be used to describe the appropriate boundary required for either surgical removal of a tumor or radiation treatment [89, 90].

Even though many treatment protocols exist, the probability of individuals surviving advanced stages of cancer is very low. As a result, many questions still remain as to how chemotherapeutic drugs work at a molecular level (e.g., which proteins they target by design and which by accident), and how radiation treatments should be delivered in order to maximize the death of cancer cells, while minimizing the harm to normal healthy tissue. Mathematical and computational modeling plays an important role in understanding radiation treatment protocols. For example, many models have been developed to define a tumor control probability (TCP) [91], where TCP is defined as the probability that no clonogenic cells survive radiation treatment [92]. TCP models have been incorporated into existing cancer models that describe cancer growth without treatment, such as those described above, to better understand the effects of radiation treatment on normal tumor dynamics [93].

The models discussed above describe the spatial and temporal changes of certain quantities that are of interest in various biological systems. In particular, the differential equations described above give temporally dependent solutions (and spatially dependent solutions in the case of the PDEs described) for various quantities, including the total populations of individuals, the total number/density of cells, or the total molecular concentration of a certain compound. Many of the successes and limitations of a differential equation modeling approach are highlighted above. One limitation, not highlighted in the above sections, is that such methods (those that use only a handful of differential equations) are not appropriate for describing the smaller scale movements of molecules. The movement and structure of an individual molecule is based on the many complex interactions between the individual atoms within a molecule, as well as its interactions with surrounding molecules. In order to follow the motions of every atom and molecule over extremely small timescales, computational methods such as molecular dynamic simulations (designed to solve extremely large systems of differential equations over very small timescales) can be applied. This technique is described in the following section.

## Molecular dynamics

The sophistication of the model used to study a given system depends on the property of interest. Often, a 3D model of a molecule or complex that shows the spatial relationships between atoms is the best way to understand a system. Such computational models provide a means of observing the structure and motion of individual atoms within complex biomolecular systems. Although a physical model of a small molecule with less than 20 atoms can be easily made from plastic or wire in a few minutes, a similar model of a protein or an enzyme involves hundreds or thousands of atoms. Over the last decade improvements in a combination of computer graphics programs, and molecular modeling techniques and hardware have resulted in an unprecedented power to create and manipulate 3D models of molecules.

**Figure 1:**
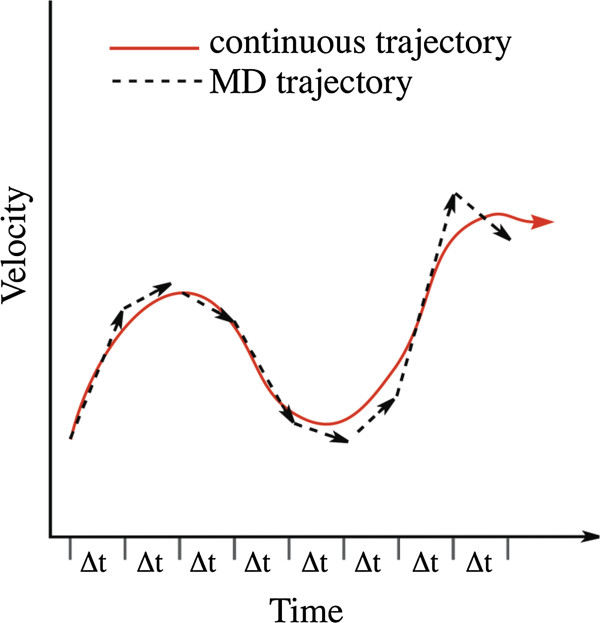
**The evolution of a trajectory, showing the continuous, true trajectory (red), being closely followed by the MD trajectory (black).**

Molecular dynamics (MD) simulations follow the motions of atoms and molecules, and provide a means of investigating biological problems at a molecular level. This is achieved by solving Newton’s equations of motion (EOM) for interacting atoms and evolving a system through time and space. Changes in atomic positions and velocities over time, usually ranging from nano- to milliseconds, result in a trajectory. In simple cases with few atoms, analytic solutions to Newton’s EOM can be obtained, giving a trajectory that is a continuous function of time (Figure 1). However, in a computer simulation with many atoms, the EOM are solved numerically. Forces are evaluated for discrete intervals, or time steps (Δ*t*, Figure 1) on the order of femtoseconds, where the forces are considered constant over a given time step. The goal is to follow the continuous function of time as closely as possible, which requires small time steps to ensure the motions of all atoms are resolved.

The forces on each atom are derived from the potential energy of the system, which can be described with quantum or classical mechanics. Since quantal descriptions are generally limited to small systems, classical descriptions are commonly used when studying biological systems and will be discussed in this section. It is worth noting that MD is a deterministic approach for exploring the potential energy surface of a system, while a stochastic approach can be obtained using Monte Carlo methods.

MD trajectories are analyzed to obtain information about the system, including structural changes as measured by atomic root-mean-square deviation (RMSD), non-covalent interactions, binding free energies [94], structural stability, short-lived reaction intermediates [95], conformational changes, flexibility, ligand binding modes [96], as well as ionic conductivity and mobility [97]. Numerous and diverse applications include the investigation of clinically important proteins such as HIV-1 gp120 [98], protein binding sites [99], drug resistance mechanisms of HIV-1 protease [100], protein folding [101, 102] and the role of crystal water molecules in ligand-protein binding [103].

### Force fields for protein simulations

Newton’s second law, , establishes the relation between mass (*m*) and acceleration (), as well as force (- ∇*V*), which is the negative of the gradient of the potential energy function (*V*). During an MD simulation, the forces acting on each atom of the system are calculated and atoms are moved according to those forces. In a classical MD simulation, the potential is calculated with a force field. The potential terms in a force field are a sum of contributions due to covalently-bonded interactions (*V*_bond_, *V*_angle_, *V*_torsion_) and non-bonded interactions. Non-bonded interactions are calculated pairwise between two atoms, denoted *i* and *j*, and commonly include van der Waals (*V*_*ij,*vdW_) and electrostatic (*V*_*ij*,electrostatic_) contributions.
30

Due to the pairwise calculation of non-bonded interactions, such force fields scale as *N*^2^, where *N* is the number of atoms in the system. Each potential term contains parameters, where fitting to experiment is required to calculate these interactions [104]. Parameter fitting is done to reproduce the behavior of real molecules. This includes determining the van der Waals’ radii, partial charges on atoms, bond lengths, bond angles and force constants. These parameters, along with the functional form of each potential term, collectively define a force field. Today, several types of force fields are available: (a) all-atoms force fields (parameters are considered for every atom), (b) united-atoms force fields (aliphatic hydrogen atoms are represented implicitly) and (c) coarse-grained force fields (groups of atoms are treated as super atoms). For a list of all force fields discussed, see Table 1.

**Table 1:** **List of force fields**

Name	Family	Type	Reference
ff94	Amber	All-atoms	[104]
ff99	Amber	All-atoms	[107]
ff99SB	Amber	All-atoms	[106]
ff03	Amber	All-atoms	[112, 113]
ff03.r1	Amber	All-atoms	
ff03ua	Amber	United-atoms	[114]
ff12SB	Amber	All-atoms	[110]
ff14SB	Amber	All-atoms	
GAFF	Amber	All-atoms	[115]
CHARMM	CHARMM	United-atoms	[117]
CHARMM19	CHARMM	United-atoms	[118]
CHARMM22	CHARMM	United-atoms	[119]
CHARMM22/CMAP	CHARMM	United-atoms	[120]
CHARMM27	CHARMM	United-atoms	[116]
CGenFF	CHARMM	United-atoms	[121]
OPLS-UA	OPLS	United-atoms	[117]
OPLS-AA	OPLS	All-atoms	[122]
GROMOS (A-version)	GROMOS	United-atoms	[125]
GROMOS (B-version)	GROMOS	United-atoms	[125]
43A1	GROMOS	United-atoms	[126]
45A3	GROMOS	United-atoms	[127]
53A5	GROMOS	United-atoms	[128]
53A6	GROMOS	United-atoms	[128]
54A7	GROMOS	United-atoms	[129, 130]
54B7	GROMOS	United-atoms	[129, 130]
54A8	GROMOS	United-atoms	[129, 130]
MARTINI	MARTINI	Coarse-grained	[131–134]

Most all-atom force fields for proteins use relatively simple functions for modeling the potential energy surface [105], which correspond to the terms in Equation (30):
31

**Figure 2:**
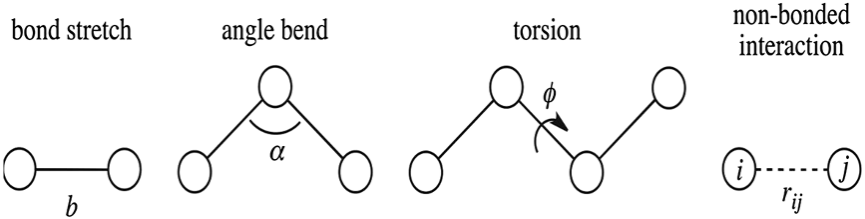
**An illustration of the variables involved in a basic all-atom force field, corresponding to Equation (**31**).**

The variables are indicated in Figure 2. The first term includes the stretching of bonds, where the potential is calculated using parameters *K*_*b*_ (related to force constant) and *b*_0_ (related to equilibrium bond length), as well as the variable, *b*, which is the distance between bonded atoms. Similarly, the second term includes angle bending, where parameters *K*_*α*_ and *α*_0_, represent force constants and equilibrium angles, and the variable (*α*) is the angle obtained from the structure of interest. The third term, representing the potential from dihedral angles, involves parameters *K*_*ϕ*_, (barrier for rotation), *n* (number of maxima) and *φ*_0_ (angular offset), as well as the variable *φ*, obtained from dihedral angles in the structure. The final two terms in Equation (31) represent non-bonded interactions between atoms *i* and *j*, which are summed over *N* atoms of the system. In this potential function, the van der Waals term is represented by the Lennard-Jones potential, where *A*_*ij*_ and *B*_*ij*_ are atom specific parameters related to atom size, and *r*_*ij*_ is a variable representing the distance between atoms *i* and *j*. The electrostatic term in Equation (31) is calculated by a Coulomb potential, where parameters *q*_*i*_ and *q*_*j*_ are (fixed) charges on atoms *i* and *j*, respectively, and the constant *ϵ*_0_ is the permittivity of free space. The force constants are obtained by empirical methods or by quantum mechanics calculations, depending on the force field. Here we present a summary of the most commonly used force fields in MD simulations, namely Amber, CHARMM, OPLS, GROMOS and MARTINI.

#### Amber force fields

The functional form from which most of the Amber (Assisted Model Building with Energy Refinement) force fields come uses Equation (31) and was developed by Cornell and co-workers (denoted ff94) [104]. However, various revisions have since been developed that vary in their parameterization, with the goal of improving results. One notable revision of Amber for proteins and nucleic acids is ff99SB [106], which was developed at Stony Brook University as a modification of the old ff99 force field [107] and improves on ff99 in its description of the φ and ψ dihedral angles of the protein backbone, resulting in a better balance between secondary structures, and improved treatment of glycine [108, 109]. The ff12SB revision [110] reparameterizes backbone torsion angles, side chain torsions in select amino acids, and incorporates improved backbone torsions in DNA and RNA, with recent studies finding ff12SB performs better than ff99SB [111]. The current and most recent version, ff14SB, is recommended by Amber developers and minimizes dependencies of protein side chain conformations on backbone conformations by including side-chain corrections, and improves upon dihedrals in DNA and RNA, particularly *χ*. Another extensively-used Amber force field is ff03 [112, 113] (the latest version is ff03.r1), which improves upon the charges calculated by ff99 by using charges derived from quantum calculations with a continuum dielectric to simulate the solvent polarization. A united-atom version of ff03, ff03ua, is also available [114]. Expanding the utility of the Amber force fields beyond peptides and nucleotides is the general Amber force field (GAFF) [115], which includes a complete set of parameters for a large number of small molecules while still remaining fully compatible with the other versions of the Amber force fields discussed above. This allows for Amber and GAFF to be combined to examine protein-ligand complexes, as well as modified proteins, DNA or RNA.

#### CHARMM force fields

The CHARMM (Chemistry at Harvard Macromolecular Mechanics) force fields are also a prominent set of force fields for studying biological systems. The functional form of the force field used by CHARMM is based on Equation (31), but also includes additional terms to treat improper torsions and atoms separated by two bonds (Urey-Bradley term) [116]. The CHARMM force fields use classical (empirical or semi-empirical) and quantum mechanical (semi-empirical or *ab initio*) energy functions for different types of molecular systems. They include parameters for proteins, nucleic acids, lipids and carbohydrates, allowing simulations on many common biomolecules. The initial version of the CHARMM force field was developed in the early 1980s, and used an atom force field with no explicit hydrogens [117]. However, in 1985, CHARMM19 parameters were developed in which hydrogen atoms bonded to nitrogen and oxygen were explicitly represented; hydrogens bonded to carbon or sulfur were still treated as extended atoms [118]. CHARMM19 parameters aimed to provide a balanced interaction between solute-water and water-water energies. Although this force field was tested primarily on gas-phase simulations, it is now used for peptide and protein simulation with implicit solvent models. Newer versions of CHARMM, such as CHARMM22, include atomic partial charges that are derived from quantum chemical calculations of the interactions between model compounds and water [119]. Although CHARMM22 is parameterized for the TIP3P explicit water model, it is frequently used with implicit solvents. CHARMM27 parameters were developed for nucleic acids (RNA, DNA) and lipid simulations [116]. Since both CHARMM27 and CHARMM22 are compatible, it is recommended CHARMM27 be used for DNA, RNA and lipids, while CHARMM22 should be applied to protein components [116]. A more recent, dihedral-corrected version of CHARMM22 was developed, denoted CHARMM22/CMAP, which improves the parameters describing the *φ* and *ψ* dihedral angles of the protein backbone [120]. A general version of the CHARMM force field (CGenFF) also exists which allows to the treatment of drug-like while maintaining compatibility with other the CHARMM force fields [121].

#### OPLS force fields

For the OPLS (Optimized Potentials for Liquid Simulations) family of force fields the form of the potential energy function differs from Equation (28) in the dihedral term, and was parameterized by Jorgensen and co-workers [117]. OPLS force fields were parameterized simulate the properties of the liquid states of water and organic liquids [118]. For proteins, a united-atom version was first developed (OPLS-UA), followed by an all-atoms version (OPLS-AA) [122]. Charges and van der Waals terms were extracted from liquid simulations. The OPLS-AA force field uses the same parameters as the Amber force fields for bond stretching and angles. The torsional parameters were obtained by using data from *ab initio* molecular orbital calculations for 50 organic molecules and ions [123]. Several improvements and re-parameterizations were proposed in later years for this set of force fields [122, 124], including for simulations of phospholipid molecules [125].

#### GROMOS force fields

The GROMOS (Groningen Molecular Simulation) force fields are united-atom force fields that that were developed in conjunction with the software package of the same name to facilitate research efforts in the field of biomolecular simulation in a university environment [125]. Its functional form varies from Equation (31) in the dihedral term, and differs from other force fields in its goal of reproducing enthalpies of hydration and solvation. The initial GROMOS force field (A-version) was developed for applications to aqueous or apolar solutions of proteins, nucleotides and sugars. However, a gas phase version (B-version) for the simulation of isolated molecules is also available [125]. Important versions of the GROMOS force fields include GROMOS 43A1 [126] (improved treatment of lipid bilayers), GROMOS 45A3 [127] (relevant to lipid membranes and micelles) and GROMOS 53A5 and 53A6 [128] (recommended for the simulation of biomolecules in explicit water). Recent releases such as GROMOS 54A7 and 54B7 involve modifications to *φ* and *ψ* protein dihedral angles, new lipid atoms types, new ion parameters and additional improper dihedral types, while GROMOS 54A8 accurately models the structural of lipid bilayers, proteins and electrolyte solutions [129, 130].

#### MARTINI force fields

The semi-empirical MARTINI force field is the most commonly used coarse-grained force field for biomolecular system simulations; it was originally developed for lipid simulations [131]. In this potential energy surface, four heavy atoms in a molecule are considered as a single interaction site, and only four types of interaction have been considered, namely polar, non polar, apolar and charged; moreover, each particle has different subtypes for taking in account the underlying atomic structure; a special particle types has been introduced for the ring conformations, as the four-to-one mapping is not appropriate to represent small ring molecules. Initially developed for lipid simulations, this force field includes now extensions for the parameterization of proteins [132], carbohydrates [133] and more recently glycolipids [134]. With the approximations introduced by the coarse grain approach it is possible to increase both the timescale and the size of systems compared with all-atoms or united-atoms force field simulations. The list of fields where MARTINI was employed includes characterization of lipid membranes [135], protein-protein interactions [136], self assembly of peptides and proteins [137] and interactions between nanoparticles and biological molecules, as for example the study of the mechanism by which fullerene can penetrate the cellular lipid membrane performed by Wong-Ekkabut et al. [138] and other applications [139]. However, MARTINI cannot be used for protein-folding studies [139], as the secondary structure is a required input parameter.

### Calculation of solvation free energies

The calculation of solvation free energies is a challenging problems in MD simulations. Determining solvation free energy is especially difficult in aqueous bio-systems due to the size of the system [140]. Solvation free energy, Δ*G*_solv_, is a thermodynamic property defined as the net energy change upon transferring a molecule from the gas phase into a solvent with which it equilibrates [141]. Solvation effects can change the physical and chemical properties of biomolecules including charge distribution, geometry, vibrational frequencies, electronic transition energies, NMR constants and chemical reactivity [142].

Several methods have been developed for modeling solvation and one can select the most advantageous choice among them based on the required accuracy and computational cost. To simulate effects of solvent on biomolecules, one can use explicit or implicit solvent models. While explicit solvent models include solvent molecules in the system, implicit models use a mean field approach [143, 144]. Although explicit solvent simulations are computationally expensive because of the enormous numbers of atoms involved, they provide a more realistic picture of solute-solvent interactions, reflecting the molecular complexity of the biomolecule and its environment. In comparison, implicit solvent models increase the speed of the simulation since the Newtonian equations of motion are not solved for additional solvent molecules. Table 2 lists the solvation models discussed.

**Table 2:** **List of solvation models**

Name	Type	Reference
Poisson–Boltzmann (PB)	Implicit	[145, 146]
Generalized Born (GB)	Implicit	[140, 141, 147, 149–151]
3D-RISM	RISM	[140, 152–158]
3D-RISM-KH	RISM	[156, 164]
MTS-MD/OIN/ASFE/3D-RISM-KH	RISM	[149]
3D-RISM-KH-NgB	RISM	[168]
SPC	Explicit	[174]
SPC/E	Explicit	[174]
POL3	Explicit	[175]
TIP3P	Explicit	[176]
TIP3P/F (TIP3P-PME/LRC)	Explicit	[177]
TIP4P	Explicit	[176, 178]
TIP4P/Ew	Explicit	[179]
TIP5P	Explicit	[180]

### Implicit water models

The simplest approach to solvation is to treat the effects (e.g. electrostatic interactions, cavitation, dispersion attraction and exchange repulsion) of solvent on the solute with an implicit model. These methods represent the solvent as a continuum environment, where the quality of the results is most affected by the electrostatic and cavitation (the size and shape of a cavity that the solute occupies) contributions. The solvation free energy of a molecule, Δ*G*_solv_, can be divided into two parts: electrostatic (Δ*G*_el_) and non-electrostatic (Δ*G*_nonel_). The electrostatic energy is defined as the free energy required to remove all the charges in vacuum and add them back to the solute in the presence of continuum solvent [140, 141]. The origin of the non-electrostatic energy is a combination of favorable solute-solvent van der Waals interactions and the unfavorable disruption of the water structure by solute molecules (cavitation), and corresponds to solvating the neutral solute. There are several different implicit solvent models discussed below: the Poisson–Boltzmann model and the generalized Born model, which differ in how Δ*G*_el_ is obtained.

#### Poisson–Boltzmann model

Solving Poisson’s equation, which is valid under conditions where ions are absent, gives a second order differential equation describing the electrostatic environment that is modeled with a dielectric continuum model [145],
32

where *φ*(*r*) is the electrostatic potential, *ϵ*(*r*) is the dielectric constant and *ρ*(*r*) is the charge density.

Poisson’s equation cannot be solved analytically for most systems, and must be solved using computers and adopting numerical methods. The Boltzmann contribution, along with the assumptions of the Debye–Hückel theory, describes the charge density due to ions in solution. This results in the (non-linearized) Poisson–Boltzmann (PB) equation [146]:
33

where *κ* denotes the Debye–Hückel parameter, ϵ_s_ is the solvent dielectric constant, *S*(*r*) is a “masking” function with value 1 in the region accessible to the ions in the solvent and value 0 elsewhere; *e* is the protonic charge; *k* is Boltzmann’s constant; *T* is the absolute temperature. Here, the charge density on the right represents the partial charges in the cavity. When the ionic strength of the solution or the potential is low, Equation (33) can be linearized by expanding the second term on the left into a Taylor series and retaining only the first term:
34

The non-electrostatic contribution (Δ*G*_nonel_) to the solvation free energy is calculated by empirical methods and is proportional to the solvent accessible surface area. This is added to the electrostatic part to yield the solvation free energy. Although the PB approach is mathematically rigorous, it is computationally expensive to calculate without approximations [141, 147, 148]. The generalized Born model provides a more efficient means of including solvent in biomolecular simulations.

#### Generalized born model

The generalized Born (GB) model is based on the Born approximation of point charges, modeling solute atoms as charged spheres with an internal dielectric (generally equal to 1) that differs from the solvent (external) dielectric. The polarization effects of the solvent are represented by a dielectric continuum represents the polarization effects of the solvent. Numerical methods are used to determine the charges on the solute spheres that result in the same electrostatic potential on the cavity surface that mimics that of the solute in a vacuum.

By making approximations to the linear Poisson-Boltzmann equation (Equation 31), the electrostatic contribution of the generalized Born model is obtained:
35

where *α*_*i*_ is effective Born radius of particle *i*, *r*_*ij*_ is the distance between atoms *i* and *j*, *ϵ*_int_ and *ϵ*_ext_ the internal and external dielectric constants, respectively, and *q*_*i*_ is the electrostatic charge on particle *i*. Like the PB method, Δ*G*_nonel_ is calculated from the solvent-accessible surface area [140, 141, 147, 149–151].

#### Reference interaction site model

Another type of solvation model is a probabilistic method known as the 3D reference interaction site model (3D-RISM) [140, 152–158]. This molecular theory of solvation simulates the solvent distributions rather than the individual solvent molecules. However, the solvation structure and the associated thermodynamics are obtained from the first principles of statistical mechanics.

In this method, the 3D site density distributions of the solvent are obtained, which accounts for different chemical properties of the solvent and solute. These properties include hydrogen bonding, hydrophobic forces and solvation thermodynamics, such as the partial molar compressibility and volume. In addition, the solvation free energy potential and its energetic and entropic components can be calculated. The solvation free energy is calculated from the RISM equation as well as the closure relation [159–163].

Several additional advances have been made in formulating improved versions of the 3D-RISM theory including the hypernetted chain (HNC) closure approximation [152, 153]. Another derivation came from the molecular Ornstein–Zernike integral equation [163] for the solute-solvent correlation functions [155, 156, 164]. Sometimes the calculated solvation free energy for ionic and polar macromolecules involves large errors due to the loss of long-range asymptotics of the correlation functions. Work has been done to account for the analytical corrections of the electrostatic long-range asymptotics for the 3D site direct correlation functions as well as the total correlation functions [157, 158, 164]. Other developments include the closure approximation, 3D-RISM-KH closure, for solid-liquid interfaces, fluid systems near structural and phase transitions, as well as poly-ionic macromolecules [156, 164].

Two methods have been developed to couple 3D-RISM with MD. The first method makes use of a multiple time step (MTS) algorithm [165, 166] wherein the 3D-RISM equations are solved for a snapshot of the solute conformation, then solved again after a few MD steps. This method is limited by the requirement to re-solve the 3D-RISM equations every few MD steps, which is computationally expensive for large biomolecular systems. The second of these methods involves the contraction of the solvent degrees of freedom and the extrapolation of the solvent-induced forces. These methods are aimed at speeding up the calculations, which is useful for larger systems [149].

Other work has involved the development of the multi-scale method of multiple time steps molecular dynamics (MTS-MD) in a method referred to as MTS-MD/OIN/ASFE/3D-RISM-KH [149]. Specifically, this method converges the 3D-RISM-KH equations at large outer time steps and uses advanced solvation force extrapolation to calculate the effective solvation forces acting on the biomolecule at inner time steps. The integration between the inner and outer time steps is stabilized by the optimized isokinetic Nosé–Hoover chain (OIN) ensemble, which enables an increase of the outer time step. Furthermore, effort was expended on MTS-MD aimed at converging the 3D-RISM-KH integral once every few OIN outer time steps, and the solvation forces in between were obtained by using solvation force-coordinate extrapolation (SFCE) in the subspace of previous 3D-RISM-KH solutions [167]. Another developed model is the 3D-RISM-KH-NgB [168]. In this model the non-polar component of the hydration free energy obtained from 3D-RISM-KH is corrected using a modified Ng bridge function [169]. Calibration of this model is based on the experimental hydration free energy values of a set of organic molecules.

Lastly, work has been done to improve the performance of 3D-RISM calculations by running them on graphical processing units (GPUs). To overcome memory issues, a modification of the Anderson Method [170] that accelerates convergence was introduced [171]. This method was reported to be eight times faster on an NVIDIA Tesla C2070 GPU as compared to the time taken on an eight-core Intel Xeon machine running at 3.33 GHz.

Although 3D-RISM calculations are more computationally expensive than GB and PB based solvation methods [172], they overcome some of the inherent shortcomings of these empirical methods [173].

#### Explicit water models

Explicit solvation is characterized by modeling individual water molecules around a solute. Several explicit water models are available in the Amber, NAMD and Gromacs MD simulation packages. These include simple explicit solvent models (SPC [174], SPC/E [174]), polarizable models (such as POL3 [175]), and fixed-charge explicit solvent models (TIP3P [176], TIP3P/F [177], TIP4P [176, 178], TIP4P/Ew [179] and TIP5P [180]).

Examples of explicit water models are the simple point charge (SPC) model and the extended simple point charge (SPC/E) model [174]. In both of these models the water molecules are rigid. A derivative of SPC with flexible water molecules has been developed [181]. Another simple explicit model is the POL3 water model, which is a polarizable model [175].

More complex explicit water models include the transferable intermolecular potential n point (TIPnP), where n represents the number of interaction sites on each model. These are the most common classes of explicit solvent models in use [147]. In the case of TIP3P, the most simple TIPnP model, the interaction sites includes the oxygen and two hydrogen atoms [176]. A re-parameterized model of TIP3P is the TIP3P-PME/LRC, also referred to as TIP3P/F [177], which calculates electrostatic contributions by particle mesh Ewald (PME) summation and includes a long-range van der Waals correction (LRC). TIP4P [176, 178] introduced a fourth dummy atom bonded to the oxygen to improve the electron distribution in the water molecule. This model has been re-parameterized for use with Ewald sums: TIP4P/Ew [176, 178]. The five interaction points in the TIP5P [180] model include two dummy atoms near oxygen, which further improves the charge distribution around the water molecule.

### Molecular dynamics methods

Examining the dynamics of a system at an atomic scale requires beginning with a model having atomic-level resolution. For biological macromolecules, this may be experimentally obtained from nuclear magnetic resonance (NMR) spectroscopy or X-ray crystallographic data. Although electron microscopy data does not provide structures with atomic resolution, this data can be combined with structural data from NMR or crystallography to obtain a high-resolution structure. NMR, crystallographic and electron microscopy structures of bio-macromolecules can be downloaded from the Protein Data Bank (http://www.pdb.org). In the absence of experimental data, homology modeling may be used to generate the 3D structure of a protein (the target) using its amino acid sequence and an experimentally-available 3D structure of a homologous protein (the template). Homology modeling can produce high-quality structures of a target protein if the sequence identity of the target and template is sufficiently high (typically > 40%).

As with any computational modeling, performing MD simulations requires a balance between accuracy and efficiency. Ideally, a system should be allowed to evolve in time indefinitely so that all states of a system may be sampled. However, this is not possible in practice. Several techniques have been developed so improve simulation efficiency, while still maintaining accuracy. During a simulation, costly force calculations are performed in discrete time increments, known as time steps, typically on the order of femtoseconds. Although it is necessary to have small time steps to properly resolve the motion of atoms, this results in many force calculations and therefore large computational costs. To address this, one can restrain the fastest vibrations, which involve hydrogen, giving the SHAKE algorithm [182]. This allows a larger time step to be used during simulations [183]. Additionally, the number of time-consuming non-bonded potentials calculated can be limited by using a cut-off based method [184]. Here, interactions are calculated between pairs of atoms within the cutoff distance, but neglected for atom pairs that are far apart. Although this is generally appropriate for the short-range nature of van der Waals interactions, using cutoffs to calculate long-range Coulomb interactions leads to instabilities [185]. Alternatively, the particle mesh Ewald (PME) approach [185–188] can be used to calculate electrostatic potentials, which involves the calculation of the short range electrostatic component in real space and the long-range electrostatic component in Fourier space.

The simplest way to enhance sampling during an MD simulation is to increase the time duration of a simulation, which is usually on the order of nano- to milli-seconds. Researchers may also conduct multiple simulations that begin with the same initial structures [189], providing denser sampling of the conformational space by utilizing multiple trajectories. Advanced techniques also exist to increase the number of states of a system that are sampled during an MD run [190]. Enhanced sampling can be achieved by metadynamics [191], which allows for the high-energy regions between minima to be explored. This helps the system to escape local free energy minima and explore metastable states separated by large free energy barriers [192]. Metadynamics, which is used to calculate static properties, can also be used to calculate dynamic properties by introducing a history-dependent biasing potential as a function of a few collective variables. Selection of collective variables is an essential part of a metadynamics run as these variables help sample different energy basins. The use of metadynamics, which is a combination of ideas involving coarse-grained dynamics in space and the introduction of a history-dependent bias, can overcome the problem of limited time scale exploration by existing sampling algorithms and computational resources. Replica-exchange molecular dynamics (REMD) has also been preferred over standard MD to enhance sampling by allowing systems of similar potential energies to sample conformations at different temperatures [193, 194]. This overcomes the energy barriers on potential energy surfaces and helps explore more conformational space. REMD can effectively sample energy landscapes, including both high- and low-energy structures, which is especially important in the case of protein folding and unfolding processes [195].

In addition to obtaining structural information about a system, molecular dynamics provides information about energetics, particularly binding. Given the inherent flexibility of the these biomolecules over time, and throughout an MD simulation, properties such as the free energy are calculated as the time-average of an ensemble of snapshots obtained from MD trajectories [196–198]. Binding calculations have applications in drug design, protein-protein interactions and DNA stability. For example, the binding free energy of a ligand to a protein is calculated as the difference in free energy between the complex, and the receptor and ligand (Δ*G*_bind_ = *G*_complex_ - *G*_receptor_ - *G*_ligand_) [199]. The free energy is calculated as a sum of the energy contributions from the force field, free energy of solvation and entropy (*G* = *E*_MM_ + *G*_sol_ + *T*Δ*S*). Commonly in MD, the solvation free energy is obtained from an implicit solvent model (Poisson-Boltzmann, generalized Born) and the solvent accessible surface area (denoted MM-PBSA and MM-GBSA, respectively) [199]. Decomposition of the binding free energies provides a means of obtaining information about the residues that significantly contribute to the binding affinity of a ligand. Pairwise decomposition may also provide insight into changes in binding that result from mutations, especially single point mutations [200].

Molecular dynamics is increasingly being used to solve a host of problems [201–207]. The simulation of bio-macromolecules, especially in conjunction with solvent, is very computationally demanding. This demand is being met by the increasing power and speed of modern computers, including special purpose computers such as Graphical Processing Units (GPUs) [208]. Novel methods have been developed to enhance the exploration of conformational space. The accuracy of force fields continues to improve with recent reparameterizations. A variety of solvation models exist, which can also be used to calculate binding free energies, which are immensely important in drug discovery applications, identifying enzyme inhibitors or compounds that block protein-protein interactions. Although classical MD simulations have been proven useful in studying a variety of biomolecular systems, they are limited in their application to stable structures. The examination of systems involving chemical reactions or quantum phenomena requires treatment using quantum mechanics methods, or the combination of quantum mechanics and molecular mechanics methods (denoted QM/MM). The application of quantum methods to biomolecules is discussed in the next section.

## Quantum mechanics in biophysical modeling

Quantum mechanics (QM) calculations, being highly accurate and rigorous, are an essential tool in computational chemistry studies. Unfortunately, the prohibitive size of many biological systems has limited theoretical and computational studies of them to the realm of classical mechanics, largely utilizing non-polarizable force fields in MD simulations. This has necessarily reduced the scope of studies to conformational or structural aspects of these bio-systems, rather than more complex problems such as chemical reactions or quantum phenomena (excited states and charge-transfer, for example). Although hybrid quantum mechanics/molecular mechanics (QM/MM) approaches have increased accuracy, recent improvements in software, hardware and theory have allowed for full quantum mechanical studies of biochemical systems. The QM methods and functionals discussed are listed in Table 3.

**Table 3:** **List of QM methods and functionals**

Name	Type	Reference
Hartree–Fock (HF)	*ab initio* method	[209]
Møller–Plesset (MP2)	*ab initio* method	[209]
Coupled-cluster (CC)	*ab initio* method	[209]
CCSD(T)	*ab initio* method	[209]
CCSD(T)/CBS	*ab initio* method	[220]
MNDO	Semi-empirical method	[210–212]
AM1	Semi-empirical method	[210–212]
PM3	Semi-empirical method	[210–212]
PM6	Semi-empirical method	[210–212]
OMx	Semi-empirical method	[210–212]
B3LYP	DFT functional	[214]
PBE	DFT functional	[214]
TPSS	DFT functional	[214]
DFT-D2	DFT method	[215, 218]
DFT-D3	DFT method	[215, 218]
Effective fragment potential (EFP)	Fragmentation method	[241–243]
Fragment molecular orbital (FMO)	Fragmentation method	[244]
Elongation (ELG)	Fragmentation method	[245]
Divide and conquer (DC)	Fragmentation method	[246]

## Quantum mechanics methods

The wavefunction, which contains all the information describing a quantum system, is obtained by solving the Schrödinger equation, usually in its time-independent, non-relativistic form, invoking the Born–Oppenheimer approximation.
36

This wavefunction, *ψ*, is a function of *N* electronic coordinates and depends parametrically on *M* nuclear coordinates. The coordinates of an electron are denoted *ζ*_*N*_ = (*x*_*N*_, *y*_*N*_, *z*_*N*_, *σ*_*N*_), where *x*_*N*_, *y*_*N*_, *z*_*N*_ are the spatial coordinates of electron *N*, and *σ*_*N*_ is the spin of this electron. The spatial coordinates of nucleus *M* are indicated by .

The Hartree–Fock (HF) method is the simplest *ab initio* QM method for obtaining the wavefunction, where the *N*-electron wavefunction is approximated as a product of *N* one-electron wavefunctions, expressed in a Slater determinant. However, the HF theory is insufficient in describing electron correlation. Electron correlation is included in post-Hartree–Fock *ab initio* methods such as the Møller–Plesset perturbation theory (MP2) or coupled-cluster (CC) calculations, although at increased computational cost. While HF scales formally as *K*^4^, where *K* is the number of basis functions, MP2 scales as *K*^5^, and the gold-standard CCSD(T) scales as *K*^7^. A detailed description of these methods, and many others, is available in the *Encyclopedia of Computational Chemistry*[209].

An alternative approach to including electron correlation is using semi-empirical quantum methods, where expensive two-electron integrals in the HF method are eliminated and the method is supplemented with parameters derived from experimental data. Although these methods are computationally very efficient, their applicability is generally limited to systems similar to the parameterization set. Many variants of semi-empirical methods exist, with applicability to biological systems [210–212], including MNDO, AM1, PM3, PM6, and the OMx methods.

The electron density, a physical observable, also determines all properties of a system, and it is this fact that is utilized in the density functional theory (DFT) [213].
37

Since DFT methods scale as *K*^3^, they are more efficient than the HF method while also containing electron correlation. Therefore, DFT has been successful in the study of a variety of biological systems [214]. Many functionals exist (for example, B3LYP, PBE and TPSS [214]), which vary based on the form of the exchange-correlation functional, and the performance of each functional is highly dependent on the system and properties of interest [213]. Even with the formal inclusion of electron correlation, many DFT functionals are inadequate in modeling dispersion forces, a dominant source of stabilization in bio-macromolecules. A common approach for improving the performance of DFT functionals is to add a dispersion correction, which can be derived either empirically or from high-level *ab initio* calculations [215–217]. For example, the DFT-D2 and DFT-D3 methods are popular [215, 218]. It is worth noting that the dispersion terms have also been applied to improve semi-empirical methods [210, 212, 217, 219].

The electronic structure methods mentioned above, particularly the *ab initio* methods, have been largely limited to studying small molecules or truncated systems of biological and medicinal/pharmaceutical interest. For example, extensive benchmarking studies have examined non-covalent interactions to identify efficient methods of comparable accuracy to CCSD(T)/CBS [220]. QM methods are also routinely used to develop MD force field parameters and charges using small models, as well as to parameterize docking scoring functions [219]. Additionally, ligand strain may be evaluated with QM methods [219]. Quantitative structure–activity relationship (QSAR) models also utilize QM methods to calculate the predictor variables such as electrostatic potentials, orbital energies, charges, and dipoles in small molecules [219, 221]. The QM methods, specifically the semi-empirical and DFT approaches, were combined with truncated protein models to examine protein-ligand interactions [211]. Nevertheless, the use of QM methods to study very large systems remains limited. In order for the extensive applications of QM methods to macromolecular systems of biological and medicinal interest to be feasible, significant advances in software, hardware, and theory must be achieved [222].

### Acceleration of quantum mechanics with graphical processing units

Graphics processing units (GPUs) have led to substantial accelerations in high-performance computing, with significant advancements for costly QM calculations. GPU-accelerated code has been developed for the HF method [223], correlated *ab initio* methods [223–225], semi-empirical methods [226, 227], and DFT [228]. In fact, GPU acceleration is now implemented in many quantum chemistry programs. NVIDIA reports the following programs as having GPU support: ABINIT, BigDFT, CP2K, GAMESS-US, GAMESS-UK, GPAW, LATTE, MOLCAS, MOPAC2012, NWChem, OCtopus, PEtot, Q-Chem, QMCPACK, Quantum Espresso, TeraChem, and VASP [208]. However, the most extensive use of GPUs in a QM code [229–234] has been implemented in TeraChem, an electronic-structure package specifically designed for use with GPUs. This has enabled a QM description of a protein to examine charge-transfer and polarization in a solvated environment [235], full QM optimizations of protein structures [233], and the examination of excitations in the green fluorescent protein (GFP) chromophore [236].

### Fragment-based quantum mechanics methods

One approach to making QM methods more tractable to biological and medicinal applications is the modification of methods so that they scale linearly with system size [237, 238]. One such approach is based on fragmentation methods, which have been developed to facilitate the application of wavefunction and density functional methods to macromolecular structures. These methods partition a macromolecular system and perform QM calculations on each fragment to obtain their wavefunctions and properties, which are then combined to arrive at properties of the macromolecular system as a whole. Fragmentation methods also benefit from their ability to be massively parallelized. A comprehensive summary of the many fragmentation methods available, with many applications to biological systems, can be found in a recent review [239].

Of the electronic structure software available, the GAMESS program includes the greatest variety of fragmentation methods [239, 240], which include the effective fragment potential (EFP) methods [241–243], the fragment molecular orbital (FMO) method [244], the elongation (ELG) method [245], and divide and conquer (DC) approaches [246]. Although the EFP methods utilize intermolecular potentials, these are distinct from those in force fields since they are rigorously derived from *ab initio* calculations rather than empirical parameters. Of these fragmentation methods, the FMO approach is arguably the most robust and has been widely applied to biological systems [239, 240, 247–249], which include drug discovery, protein-ligand binding, protein-protein interactions, enzymatic catalysis, and DNA.

### Application of quantum mechanics/molecular mechanics to computational enzymology

Enzymology investigates, among other topics, enzyme kinetics and mechanisms of inhibition in steady-state turnover. Advances in technology and methods have led to more detailed information about enzyme structures and mechanisms. With an explosion in the number of novel and uncharacterized enzymes identified from the vast number of genome sequences, it has become evident that the structural and functional properties of these enzymes need to be elucidated to establish precisely their mechanisms of action and how the enzymes fit into the complex webs of metabolic reactions found in even the simplest of organisms [250].

Vast changes have occurred in the science of enzymology since molecular simulations and modeling were first developed. Calculations can provide detailed, atomic-level insights into the fundamental mechanisms of biological catalysis. Computational enzymology was launched in the 1970s [251]. The pioneering studies of Warshel are particularly notable [252, 253]. By the early 1990s the number of computational mechanistic studies of enzymes was still relatively small [254, 255], but recently there have been a great number of computational studies of enzymatic reaction mechanisms published [253, 256–258]. Currently, computational enzymology is a rapidly developing area, focused on testing theories of catalysis, challenging “textbook” mechanisms, and identifying novel catalytic mechanisms [259].

The choice of an appropriate method for the particular enzyme being modeled is vital. Quantitative predictions of reaction rates or the effects of mutations remain very challenging, but with appropriate methods, useful predictions can be made with some confidence. Careful testing and experimental validation are important. For example, a comparison of calculated barriers for a series of alternative substrates with experimentally determined activation energies demonstrated good correlation validating mechanistic calculations [260, 261]. Some enzymes have become important model systems in the development and testing of computational methods and protocols; these include chorismate mutase [259, 262–266], citrate synthase [267–269], P450 [257, 259], para-hydroxybenzoate hydroxylase [257, 260, 265] and triosephosphate isomerase [255, 270, 271].

### Modeling enzyme-catalyzed reactions

The usual starting point for modeling an enzyme-catalyzed reaction is an enzyme structure from X-ray crystallography. When this is not available, sometimes a model may be constructed based on homology to other structures that have been solved [272], though such models should be treated with much more caution. The first step in studying an enzyme-catalyzed reaction is to establish its chemical mechanism. Its goal is to determine the functions of catalytic residues, which are often not obvious. Even the identities of many important groups may not be certain. Any specific interactions that stabilize transition states or reactive intermediates should also be identified and analyzed.

Enzymes, representing usually large molecules, need sophisticated modeling steps as the reactions that they catalyze are complex. This can be complicated further by the need to include a part of a particular enzyme’s molecular environment, such as the surrounding solvent, cofactors, other proteins, a lipid membrane, or DNA. There are many practical considerations in simulating such complex systems, such as the proper interpretation of crystal structures and the choice of protonation states for ionizable amino acids [273]. Here, we illustrate these challenges with recent examples of modeling enzyme-catalyzed reactions.

#### The empirical valence bond method

The empirical valence bond (EVB) is considered to be one of the main methods used for modeling enzyme-catalyzed reactions [274]. In the EVB method, a few resonance structures are chosen to represent the reaction. The energy of each resonance form is given by a simple empirical force field, with the potential energy given by solving the related secular equation. The EVB Hamiltonian can be calibrated to reproduce experimental data for a reaction in solution, or *ab initio* results can be used [275]. The surrounding protein and solvent are modeled by an empirical force field, using proper long-range electrostatics. The free energy of activation is calculated from free energy perturbation simulations [276]. The free energy surfaces can be calibrated by comparison with experimental data for reactions in solution. The EVB method allows the use of a non-geometrical reaction coordinate, which helps to evaluate non-equilibrium solvation effects [274]. A mapping procedure gradually moves the system from the reactants to products. The simplicity of the EVB potential function allows extensive molecular dynamics (MD) simulations, giving good sampling [277]. The EVB method has been widely used for studying reactions in condensed phases, particularly in enzymes [278–284].

#### Quantum chemical methods

Another approach to the modeling of enzyme-catalyzed reactions is to study only the active site using quantum chemical methods. This methodology is usually named the cluster approach or the supermolecule approach. The active site model should contain molecules representing the substrate(s), any cofactors, and enzyme residues involved in the chemical reaction or in binding substrates. Important functional groups are represented by small molecules (e.g., acetate can represent an aspartate side chain). The initial positions of these groups are usually coordinates taken from a crystal structure, or from an MD simulation of an enzyme complex.

Quantum chemical methods can give excellent results for reactions of small molecules. Semi-empirical techniques, such as AM1 and PM3, can model larger systems that contain hundreds of atoms. However, semi-empirical methods must be applied with caution due to their sensitivity to parameterization, because typical errors may be over 10 kcal/mol for barriers and reaction energies [285, 286]. DFT methods are considerably more accurate, while also allowing calculations on relatively large systems (e.g., active site models on the order of 100 atoms), larger than is feasible with correlated *ab initio* calculations. Many DFT methods, however, do not properly account for dispersion forces, which are important in the binding of ligands to proteins and can also be important in the calculation of energy barriers [287]. Dispersion corrections may be required in such cases [215–217]. Calculations on active site models can provide models of transition states and intermediates, which has proved particularly useful for studying metalloenzymes using DFT methods [288, 289].

#### Combined quantum mechanics/molecular mechanics methods

Combined quantum mechanics/molecular mechanics (QM/MM) methods are widely applied to accurately model enzymes. This has been made possible by increased computer power, and improved software packages. QM/MM approaches treat a small part of the system quantum mechanically (describing the electronic structure of molecules) and the rest of the system with a molecular mechanics (MM) method (using a classical potential energy function [290]). The QM treatment accounts for the electronic rearrangements involved in bond breaking and bond making, while the MM treatment allows to include the effects of the environment on the reaction energetics.

There are two general types of QM/MM methods. The first is additive,
38

where *E*_QM_(QM) is the energy of the QM region according to the QM method, *E*_MM_(MM) is the energy of the MM region according to the MM method, and *E*_QM-MM,interaction_ is the interaction energy between the two regions.

The second type of QM/MM method is subtractive,
39

where *E*_MM_ is the energy of the total system as calculated by the MM method, *E*_QM_(QM) is the energy of the QM region as calculated by the QM method, and *E*_MM_(QM) is the energy of the QM region as calculated by the MM method. This is used, for example, in the ONIOM method [291].

In the subtractive approach, the active site region is modeled at the MM level, and the choice of suitable MM parameters (e.g., atomic charges) for all states of the reaction is an important and delicate consideration. Until recently, calculations using the subtractive approach typically used the more approximate “mechanical embedding” scheme, whereas currently most implementations of both approaches allow “electrostatic embedding” [291] which takes into account the electrostatic influence of the MM region on the QM region, i.e., polarization of the QM region by the atomic charges of the MM region. Five general aspects are important in a QM/MM calculation on an enzyme:

1. Choice of the QM method.
2. Choice of the MM force field (including the MM parameters for the QM region).
3. Partitioning of the system into QM and MM regions with due attention paid to any chemical bonds that straddle the two regions.
4. Type of simulation (e.g., an MD simulation, or calculation of potential energy profiles); whether extensive conformational sampling will be performed.
5. Construction (and testing) of an accurate molecular model of the enzyme complex.

The MM force field employed in a QM/MM study should be chosen to describe the part of the system outside the QM region and its interactions with the QM region. For proteins, standard all-atom force fields such as CHARMM27, AMBER ff99 or ff99SB, and OPLS-AA are commonly used. Apart from selecting suitable QM and MM methods and a QM/MM approach, modeling an enzyme reaction with a QM/MM method requires other important choices, such as deciding which atoms to include in the QM region and how to treat covalent bonds that cross the QM/MM boundary [292]. Another important choice is determining the protonation states of residues, and how (long-range) electrostatic interactions are treated. The influence of such choices on the results should be tested [267, 293–296] in order to be able to draw reliable conclusions. Recent improvements allow relocating the QM-MM boundary on-the-fly (adaptive partitioning) [297].

### Modeling enzyme reactions by calculating potential energy surfaces

With QM or QM/MM methods, potential energy surfaces of enzyme reaction mechanisms can be explored accurately enough to enable discrimination between different mechanisms, e.g., if the barrier for a proposed mechanism is significantly larger than that derived from experiment (using transition state theory), within the limits of accuracy of the computational method and experimental error, then that mechanism can be considered to be unlikely. A mechanism with a calculated barrier comparable to the apparent experimental barrier (for that step, or failing that for the overall reaction) is more likely. However, to calculate rate constants also requires reliable estimates of enthalpies, internal energies, and free energies of a given reaction and activation, given the potential energy surface. Traditional approaches to modeling reactions rely on the identification of stationary points (reactants, products, intermediates, transition states) via geometry optimization, followed by the computation of second derivatives to enable relatively simplistic evaluation of zero-point corrections, thermal and entropy terms. Algorithms developed for small molecules are often not suitable for large systems, storage and manipulation of Hessian matrices become extremely difficult. A basic means of modeling approximate reaction paths is the “adiabatic mapping” or “coordinate driving” approach. The energy of the system is found by minimizing the energy at a series of fixed (or restrained) values of a reaction coordinate, e.g., the distance between two atoms. This approach has been successfully applied to many enzymes [257], but it is only valid if one conformation of the protein can represent the state of the system at a particular value of the reaction coordinate.

Calculations of a potential energy surface may not consider significant conformational fluctuations of the enzyme. Conformational changes, even on a small scale, may cause significant chemical changes. Conformational changes of the active site can greatly affect the energy barrier. To take the fatty acid amide hydrolase as an example, conformational fluctuations do not affect the general shape of the potential energy surfaces, but consistency between experimental and calculated barriers is observed only with a specific infrequent arrangement of the enzyme-substrate complex [298]. These findings indicate that investigation of different protein conformations is essential for a meaningful determination of the energetics of enzymatic reactions for calculations of potential energy profiles or surfaces.

### Calculating free energy profiles for enzyme-catalyzed reactions

According to transition state theory, the rate constant of a reaction is related to the free energy barrier. The techniques described previously calculate potential energy barriers for a particular conformation. Techniques that illustrate configurations along a reaction coordinate give a more sophisticated and extensive description by taking account of multiple conformations and estimating entropic effects, and can be essential for modeling enzyme reactions. Simulations of this type provide estimates of the free energy profile along a specific reaction coordinate, which is often referred to as the potential of mean force. MD and Monte Carlo methods allow such illustration, but do not provide a sufficiently detailed view of high energy regions, such as those in the vicinity of transition states. Conformational illustration of processes of chemical change requires specialized techniques, e.g., to bias the simulation to sample the transition state region. Umbrella sampling, which is widely used in MD simulations, when combined with QM/MM techniques, can be used to model enzymatic reactions [262]. QM/MM umbrella sampling simulations are possible with semi-empirical molecular orbital methods (e.g., AM1 or PM3). Often, such methods are highly inaccurate for reaction barriers and energies but their accuracy can be improved significantly by re-parameterization for a specific reaction.

## Conclusions

This review paper has aimed to provide a comprehensive guide to a plethora of mathematical and computational methods developed in the past few decades to tackle key quantitative problems in the life sciences. Our critical overview covers methods used across the life sciences, starting from macroscopic systems such as those in evolutionary biology, and ending with atomic level descriptions of biomolecules including quantum mechanical or hybrid classical/quantum approaches. Particular attention was given to large-scale computational methods, such as molecular dynamics, which play pivotal roles in the development of our understanding of molecular mechanisms at the level of molecular, structural, and cell biology. Important applications in medicine and pharmaceutical sciences have been discussed, in particular in the context of extracting crucial conclusions about complex system behavior with information limitations. We hope the reader will be encouraged to explore particular topics at a deeper level using the information and references provided in this review.

## Abbreviations

AIDS: Acquired immunodeficiency syndrome
AMBER: Assisted model building with energy refinement
AMP: Adenosine monophosphate
ASFE: Advanced solvation force extrapolation
cAMP: Cyclic adenosine monophosphate
CF: Cystic fibrosis
COPASI: Complex pathway simulator
DFT: Density functional theory
DNA: Deoxyribonucleic acid
DTI: Diffusion tensor imaging
EC: Endothelial cell
ECM: Extracellular matrix
EOM: Equations of motion
EVB: Empirical valence bond
GAMESS: General atomic and molecular electronic structure system
GB: Generalized born
GF: Growth factor
GFP: Green fluorescent protein
GPCR: G protein-coupled receptor
GPU: Graphical processing unit
GROMOS: Groningen molecular simulation
HNC: Hypernetted chain
LJ: Lennard-Jones
LRC: Long-range van der Waals correction
MARTINI: Coarse-grained force field
MD: Molecular dynamics
MM: Molecular mechanics
MM-GBSA: Molecular mechanics Generalized Born surface area
MM-PBSA: Molecular mechanics Poisson–Boltzmann surface area
MRI: Magnetic resonance imaging
MTS: Multiple time step
MTS-MD: Multiple time step molecular dynamics
NAMD: Not just another molecular dynamics software
NMR: Nuclear magnetic resonance
ODE: Ordinary differential equation
OIN: Optimized isokinetic Nosé–Hoover chain
ONIOM: Our own N-layered Integrated molecular Orbital and molecular Mechanics
OPLS: Optimized Potentials for Liquid Simulations
P450: Cytochrome P450 superfamily of monooxygenases
PB: Poisson–Boltzmann
PDB: Protein data bank
PDE: Partial differential equation
PME: Particle mesh Ewald
PMF: Potential mean force
QM: Quantum mechanics
QM/MM: Quantum mechanics/molecular mechanics
QSAR: Quantitative structure–activity relationship
REMD: Replica-exchange molecular dynamics
RISM: Reference interaction site model
RMSD: Root-mean-square deviation
RNA: Ribonucleic acid
SBML: Systems biology markup language
SBW: Systems biology workbench
SEIR: Susceptible-infected-exposed-recovered model
SEIRS: Susceptible-infected-exposed-recovered-susceptible model
SELEX: Systematic Evolution of Ligands by Exponential Enrichment
SFCE: Solvation force-coordinate extrapolation
SHAKE: Algorithm for molecular dynamics
SIR: Susceptible-infected-recovered model
SIRS: Susceptible-infected-recovered-susceptible model
TAF: Tumor angiogenic factor
TCP: Tumor control probability
XML: Extensible markup language.
